# The MRN complex and topoisomerase IIIa–RMI1/2 synchronize DNA resection motor proteins

**DOI:** 10.1016/j.jbc.2022.102802

**Published:** 2022-12-16

**Authors:** Michael M. Soniat, Giaochau Nguyen, Hung-Che Kuo, Ilya J. Finkelstein

**Affiliations:** 1 Department of Molecular Biosciences, The University of Texas at Austin, Austin, Texas, USA; 2 Center for Systems and Synthetic Biology, The University of Texas at Austin, Austin, Texas, USA

**Keywords:** DNA resection, double-strand break, single molecule, DNA curtains, BLM, DNA2, BLM, bloom syndrome helicase, BSA, bovine serum albumin, BTRR, BLM–TOP3A–RMI1/2, DSB, dsDNA break, EXO1, exonuclease 1, HA, hemagglutinin, Hd, helicase-deficient, HR, homologous recombination, MR, MRE11–RAD50, MRN, MRE11–RAD50–NBS1, MRX, Mre11–Rad50–Xrs2, Ni–NTA, nickel–nitrilotriacetic acid, QD, quantum dot, RPA, replication protein A, TOP3A, topoisomerase IIIa

## Abstract

DNA resection—the nucleolytic processing of broken DNA ends—is the first step of homologous recombination. Resection is catalyzed by the resectosome, a multienzyme complex that includes bloom syndrome helicase (BLM), DNA2 or exonuclease 1 nucleases, and additional DNA-binding proteins. Although the molecular players have been known for over a decade, how the individual proteins work together to regulate DNA resection remains unknown. Using single-molecule imaging, we characterized the roles of the MRE11–RAD50–NBS1 complex (MRN) and topoisomerase IIIa (TOP3A)–RMI1/2 during long-range DNA resection. BLM partners with TOP3A–RMI1/2 to form the BTRR (BLM–TOP3A–RMI1/2) complex (or BLM dissolvasome). We determined that TOP3A–RMI1/2 aids BLM in initiating DNA unwinding, and along with MRN, stimulates DNA2-mediated resection. Furthermore, we found that MRN promotes the association between BTRR and DNA and synchronizes BLM and DNA2 translocation to prevent BLM from pausing during resection. Together, this work provides direct observation of how MRN and DNA2 harness the BTRR complex to resect DNA efficiently and how TOP3A–RMI1/2 regulates the helicase activity of BLM to promote efficient DNA repair.

Homologous recombination (HR) is one of two major eukaryotic dsDNA break (DSB) repair pathways. HR uses the intact sister chromatid during the S/G2 phase to promote error-free repair of DSBs (1, 2). HR initiates when the resection machinery, termed the resectosome, assembles to process (resect) the genome to generate kilobase-length stretches of ssDNA (3, 4, 5, 6, 7, 8, 9, 10, 11, 12). The resectosome is a multienzyme complex composed of a helicase, a nuclease, and regulatory proteins. In humans, resection is initiated when MRE11–RAD50 (MR)–NBS1 (MRN) and CtIP make an initial incision at the DSB (11, 13). This aids in the assembly of the core resectosome consisting of the bloom syndrome helicase (BLM) along with exonuclease 1 (EXO1) or DNA2 nuclease/helicase (Fig. 1*A*) (7, 10). The resulting ssDNA produced by the resectosome is rapidly bound by the ssDNA-binding protein replication protein A (RPA), which protects the ssDNA from degradation before being replaced by the RAD51 recombinase for downstream homology search (14, 15, 16, 17).

**Figure 1:**
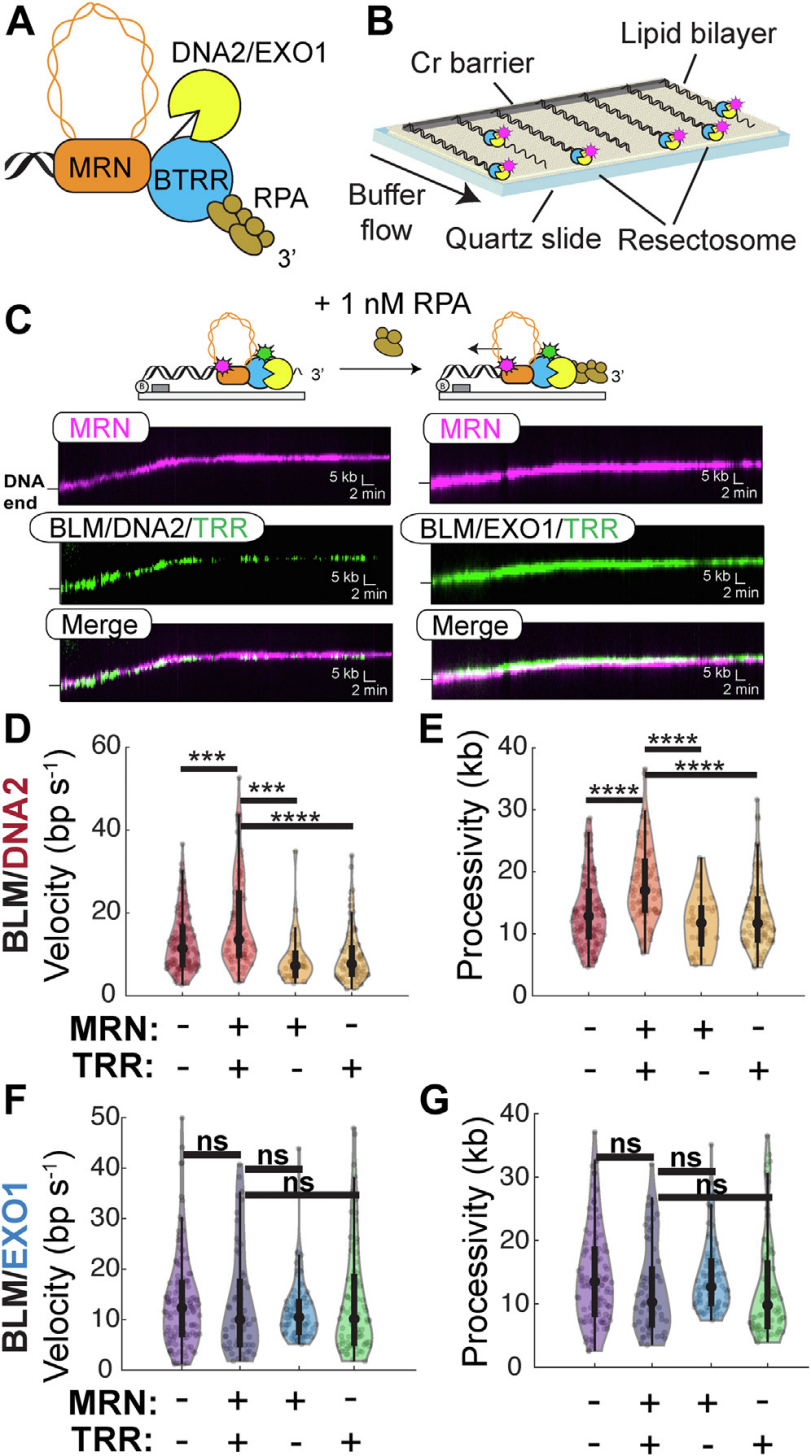
**MRN and TOP3A–RMI1/2 stimulate DNA2-mediated resection.***A*, schematic of the human resectosome, consisting of MRE11–RAD50–NBS1 (MRN; *orange*), the nucleases DNA2 or EXO1 (*yellow*), BLM–TOP3A–RMI1/2 (*blue*), and replication protein A (RPA; *brown*). *B*, schematic of single-molecule resection assay (*C*) representative kymographs of MRN (*magenta*), BTRR (*green*), and DNA2 or EXO1 resecting DNA. *D*, velocities and (*E*) processivities of BLM–DNA2 with and without TOP3A–RMI1/2 or MRN complex (n > 50 for all experiments). *Black bars* show the interquartile range (*thick bars*) and 1.5× interquartile range (*thin bars*). The *black dot* in the *middle* is the median. *F*, velocities and (*G*) processivities of BLM–EXO1 with and without TOP3A–RMI1/2 or MRN complex (n > 50 for all experiments). (Not significant; ns, *p* > 0.05; ∗∗∗∗*p* < 0.0001). BTRR, BLM–TOP3A–RMI1/2; EXO1, exonuclease 1; MRN, MRE11–RAD50–NBS1; TOP3A, topoisomerase IIIa.

Accessory proteins regulate DNA resection to both initiate in a timely manner and to prevent over-resection and subsequent loss of genetic information (8, 12, 18). For example, in *Saccharomyces cerevisiae*, the Sgs1 helicase (BLM homolog) and Dna2 nuclease/helicase interact with Mre11–Rad50–Xrs2 (MRX; the MRN homolog) and Top3–Rmi1 for efficient DNA resection (19, 20). The MRN complex has both exonuclease and endonuclease activity, which is important for initial processing and the removal of protein adducts at DSBs (21, 22, 23, 24, 25, 26). In addition to its nuclease-dependent roles, MRN/MRX also stimulates DNA resection *via* an incompletely understood nuclease-independent mechanism (19, 20, 27, 28, 29, 30, 31, 32). The human homologs of the *S. cerevisiae* Top3–Rmi1 are TOP3A (a type 1A topoisomerase) and the heterodimer RMI1–RMI2 (yeast do not encode an RMI2 homolog). These proteins interact with BLM to form the BTRR (BLM–TOP3A–RMI1/2) complex (also known as the BLM dissolvasome) (33, 34, 35, 36, 37, 38, 39). BTRR participates in DNA resection, double Holliday junction dissolution, resolution of ultrafine bridges, replication fork reversal, and interstrand crosslink repair (40, 41, 42, 43). During the initial stages of HR, the BTRR complex promotes long-range DNA2-mediated resection (44, 45). In later stages of HR, following RAD51 loading and strand invasion, BTRR is critical for the branch migration of Holliday junctions to form a hemicatenane, followed by decatenation by TOP3A–RMI1/2, resulting in noncrossovers (46, 47). Though the role of TOP3A–RMI1/2 is well defined in the later stages of HR, their precise roles in DNA resection remain unclear. For example, a catalytically inactive TOP3A mutant stimulates long-range DNA resection *in vitro*, suggesting that TOP3A–RMI1/2 may play a nonenzymatic role in resectosome assembly and/or translocation (20, 44).

Here, we use single-molecule fluorescence imaging to decipher the functions of individual resectosome components during DNA resection. Both MRN and TOP3A–RMI1/2 help BLM to initiate DNA unwinding. MRN and TOP3A–RMI1/2 also stimulate DNA2-mediated resection. Finally, MRN synchronizes the translocation speeds of BLM and DNA2 to prevent BLM pausing. We reveal that MRN and TOP3A–RMI1/2 are regulatory resectosome components that initiate DNA resection and synchronize the individual motors during kilobase-long DNA processing.

## Results

### MRN and TOP3A–RMI1/2 together stimulate the DNA2 resectosome

To understand how MRN and TOP3A–RMI1/2 regulate DNA processing, we adapted our single-molecule resection assay to quantify the movement of DNA2 or EXO1 in complex with MRN and BTRR (Fig. 1) (28, 48, 49, 50). We purified RMI1/2 with an N-terminal FLAG epitope on the RMI2 subunit. TOP3A–RMI1/2 was reconstituted by mixing TOP3A and RMI1/2 in a 1:3 ratio, followed by size-exclusion chromatography. These three proteins formed a stable complex that eluted as a single peak on a Superose 6 column (GE Healthcare) (Fig. S1*A*). TOP3A–RMI1/2 was then mixed with BLM to assemble the BTRR resectosome and labeled with fluorescent anti-FLAG antibodies (targeting RMI2 as described previously). Biotinylated MRN was conjugated with streptavidin quantum dots (QDs) that emit in spectrally distinct channels (Fig. S1, *A* and *B*). We have previously shown that fluorescently labeled MRN retains its biochemical activities, including diffusion along nucleosomal DNA and nucleolytic removal of Ku from DNA ends (28, 51, 52). In addition, QD-labeled MRN does not prevent interactions with other resectosome components, such as EXO1, consistent with *in vivo* and ensemble *in vitro* assays with unlabeled MRN. For the single-molecule resection assay, 48.5-kb-long dsDNAs with biotin on one end and a 78 nt 3′-overhang on the opposite end are organized on the surface of a microfluidic flow cell (Fig. 1*B*) (49, 53). Fluorescent BTRR complex is incubated with MRN and DNA2 or EXO1 before being injected into flow cells for single-molecule imaging (Fig. S1*C*). As expected, MRN and BTRR in complex with DNA2 and EXO1 bound the free DNA ends and resected the DNA in the presence of 1 nM RPA (Fig. 1*C*). The MRN–BTRR–DNA2 complex resected DNA for 18 ± 6 kb (mean ± SD; n = 82) with a velocity of 18 ± 11 bp s^−1^. Omitting either TOP3A–RMI1/2 or MRN decreased BLM–DNA2 velocity for approximately two-fold (BTRR–DNA2: 9 ± 6 bp s^−1^, n = 94 molecules; MRN–BLM–DNA2: 9 ± 6 bp s^−1^, n = 30) and decreased processivity by 1.4-fold (BTRR–DNA2: 13 ± 5 kb; MRN–BLM–DNA2: 12 ± 5 kb) (Fig. 1, *C*–*E* and Table S1). Our results with the human resectosome are consistent with the stimulation of *S. cerevisiae* Sgs1–Dna2 by MRX and Top3–Rmi1 (19, 20). In contrast, the addition of MRN and TOP3A–RMI1/2 to BLM–EXO1 resectosomes did not change the processivity or velocity (∼12 ± 7 kb; ∼13 ± 10 bp s^−1^; n > 50 for all conditions), suggesting that MRN and TOP3A–RMI1/2 selectively regulate DNA2-mediated resection (Fig. 1, *F* and *G* and Table S1).

### MRN and TOP3A–RMI1/2 recruit DNA2 to free DNA ends

**Figure 2:**
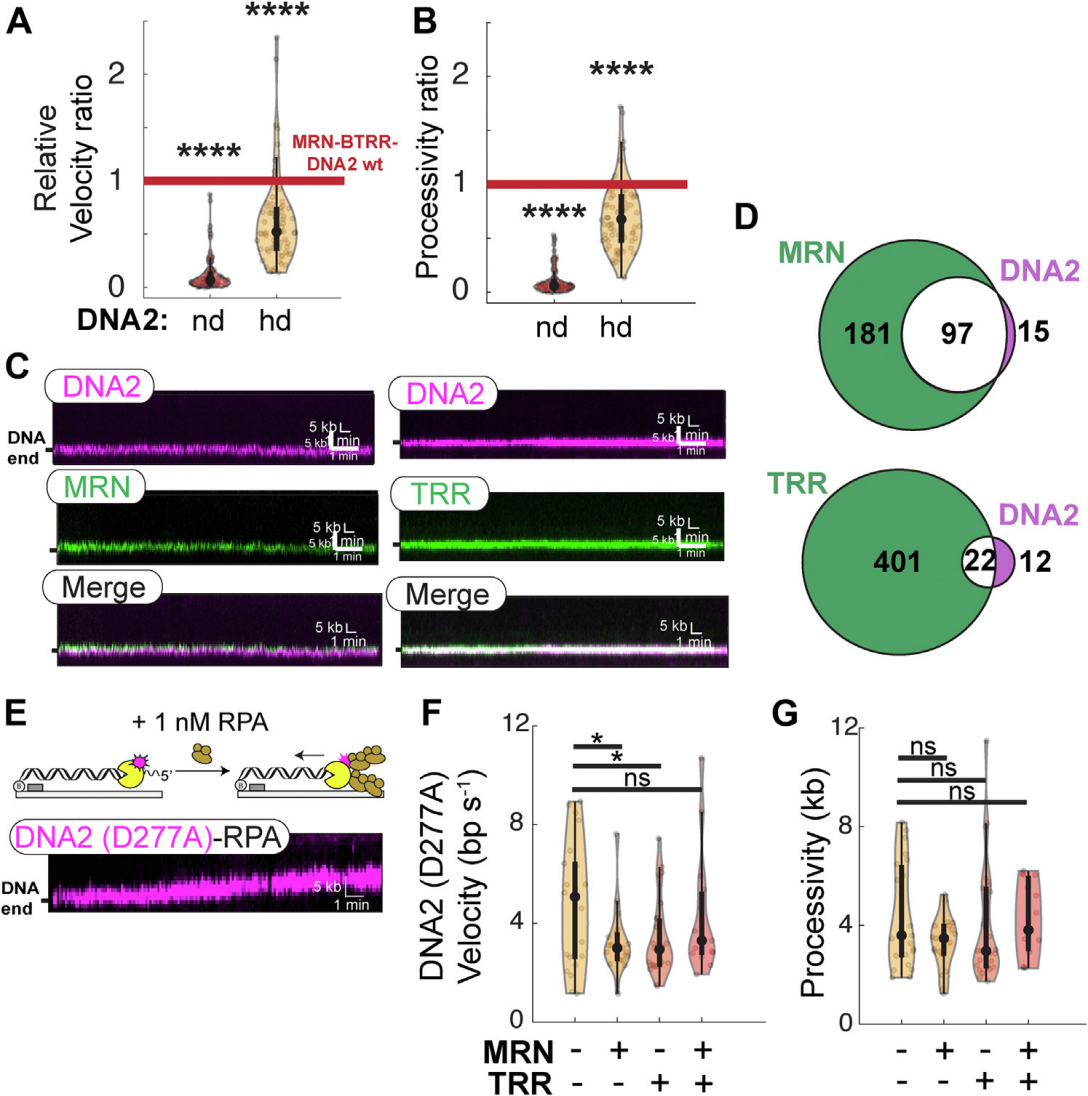
**MRN and TOP3A–RMI1/2 recruit DNA2 to free DNA ends.***A*, ratio of MRN–BTRR–DNA2 velocities and (*B*) processivities with nuclease-deficient (nd) or helicase-deficient (hd) DNA2 mutants. Both velocity and processivity are compared with the ratios of MRN–BTRR–DNA2 wildtype complex from Figure 1, *D* and *E*. (ns, *p* > 0.05; ∗*p* < 0.05; ∗∗*p* < 0.01; ∗∗∗*p* < 0.001; and ∗∗∗∗*p* < 0.0001). *C*, representative kymographs of colocalization of DNA2 (*magenta*) with MRN and TOP3A–RMI1/2 (TRR; *green*) at DNA ends. MRN and TRR were imaged in different experiments to guarantee an unambiguous fluorescent signal. *D*, Venn diagram that shows MRN and TOP3A–RMI1/2 colocalize DNA2 at free DNA ends. This interaction greatly increases the number of DNA-bound DNA2 molecules. *E*, representative kymograph showing helicase activity of nd DNA2 (D277A) mutant in the presence of RPA. *F*, velocities and (*G*) processivities of DNA2 (D277A) helicase activity with and without TOP3A–RMI1/2 or MRN complex. (ns, *p* > 0.05; ∗*p* < 0.05; ∗∗*p* < 0.01; ∗∗∗*p* < 0.001; and ∗∗∗∗*p* < 0.0001). BTRR, BLM–TOP3A–RMI1/2; MRN, MRE11–RAD50–NBS1; ns, not significant; RPA, replication protein A; TOP3A, topoisomerase IIIa.

In addition to its nucleolytic activity, DNA2 also encodes a 5’→3′ helicase domain that can unwind kilobases of dsDNA (54, 55, 56, 57, 58). We first tested the importance of both DNA2’s nuclease and helicase activity in DNA resection with MRN and the BTRR complex on the 3′-overhang DNA substrate. As expected, the nuclease-deficient DNA2(D277A) inhibited DNA resection (processivity: 2 ± 2 kb; velocity: 2 ± 2 bp s^−1^, n = 89) (Fig. 2, *A* and *B*). Furthermore, a helicase-deficient (hd) DNA2(K654R) mutant decreased resection processivity and velocity (processivity: 13 ± 6 kb; velocity: 11 ± 7 bp s^−1^, n = 76). Omitting TOP3A–RMI1/2 and MRN abrogated the negative effects of DNA2(K654R) on resection processivity and velocity (14 ± 8 kb; 12 ± 8 bp s^−1^, n = 42) (Fig. S2, *A* and *B*). We repeated the resection experiments with an hd BLM(K695A) mutant on both 5′-overhang and 3′-overhang DNA substrates but did not see long-distance motor activity beyond our ∼500 bp resolution (Fig. S2*C*). This indicates that the helicase activity of BLM provides a 5′-flap for DNA2. In addition, BLM prevents the initiation of long-range helicase activity by DNA2, even with a 12-nt 5′-overhang. These results are consistent with our previous study that showed that hd BLM also blocks nuclease-dead DNA2 from unwinding DNA (50). These results show that both the nuclease and helicase activity of DNA2 are required for rapid long-range DNA processing.

Since TOP3A–RMI1/2 and MRN stimulate the BLM–DNA2 resectosome, we tested whether either TOP3A–RMI1/2 and/or MRN can stimulate DNA2 alone. MRN recruits DNA2 to DSBs in human cells but does not affect DNA2 nuclease activity *in vitro* (30). Consistent with this report, 87% of DNA2 molecules colocalized with MRN (n = 97/112) at free DNA ends (Fig. 2, *C* and *D*). We saw a more modest 65% of TOP3A–RMI1/2 complexes (n = 22/34) colocalizing with DNA2. DNA2 did not translocate beyond our spatial resolution of ∼500 bp with MRN and TOP3A–RMI1/2 (together or independently) in the presence of RPA. Previous studies showed that suppression of the nuclease activity of DNA2 stimulates processive helicase activity on DNA substrates containing a 5′-flap in the presence of RPA (54, 55, 56). To observe the helicase activity, we monitored nuclease-deficient DNA2(D277A) on DNA substrates containing 12 nt 5′-overhang in the presence of 1 nM RPA, similar to previous studies (54, 56, 59). DNA2 was a processive helicase, motoring ∼4 ± 2 kb with a velocity of 5 ± 3 bp s^−1^ (n = 23 molecules) (Fig. 2, *E*–*G* and Table S2). The addition of MRN, TOP3A–RMI1/2, or both together had no effect on the processivity of DNA2. However, adding either MRN or TOP3A–RMI1/2 decreased the velocity of DNA2 by ∼1.4-fold relative to DNA2(D277A) alone. In contrast, the addition of both MRN and TOP3A–RMI1/2 restored helicase activity to that of DNA2 alone. These results are broadly consistent with a model where MRN and TOP3A–RMI1/2 help DNA2 engage free DNA ends but do not stimulate its nuclease or motor activities.

### TOP3A–RMI1/2 helps BLM initiate DNA unwinding

**Figure 3:**
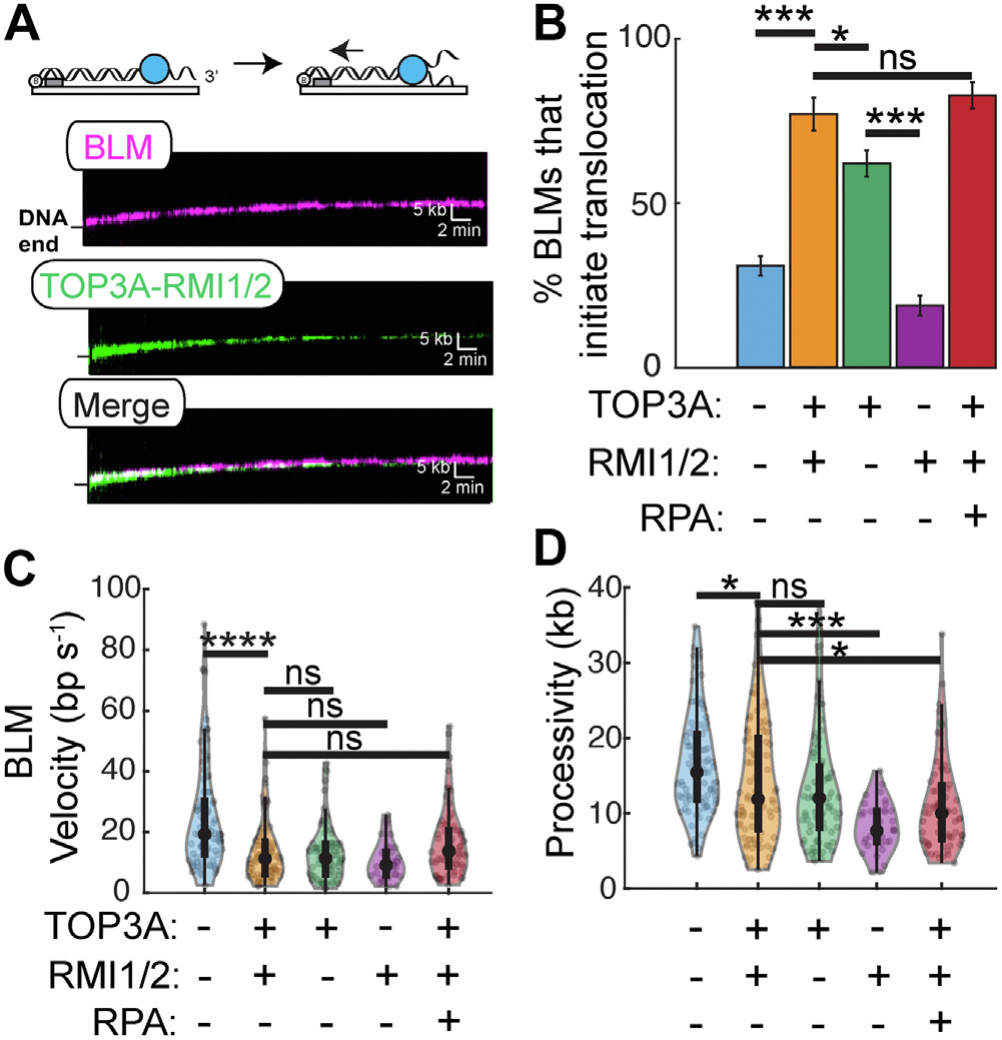
**TOP3A–RMI1/2 complex promotes initiation of BLM helicase activity.***A*, representative kymographs showing helicase activity of BLM (*magenta*) with the TOP3A–RMI1/2 complex (*green*) along DNA. *B*, TOP3A–RMI1/2 stimulates BLM helicase initiation. Error bars represent SD as determined by bootstrap analysis. *C*, velocities and (*D*) processivities of the helicase activity of the BTRR complex. (ns, *p* > 0.05; ∗*p* < 0.05; ∗∗*p* < 0.01; ∗∗∗*p* < 0.001; and ∗∗∗∗*p* < 0.0001). BLM, bloom syndrome helicase; BTRR, BLM–TOP3A–RMI1/2; ns, not significant; TOP3A, topoisomerase IIIa.

**Figure 4:**
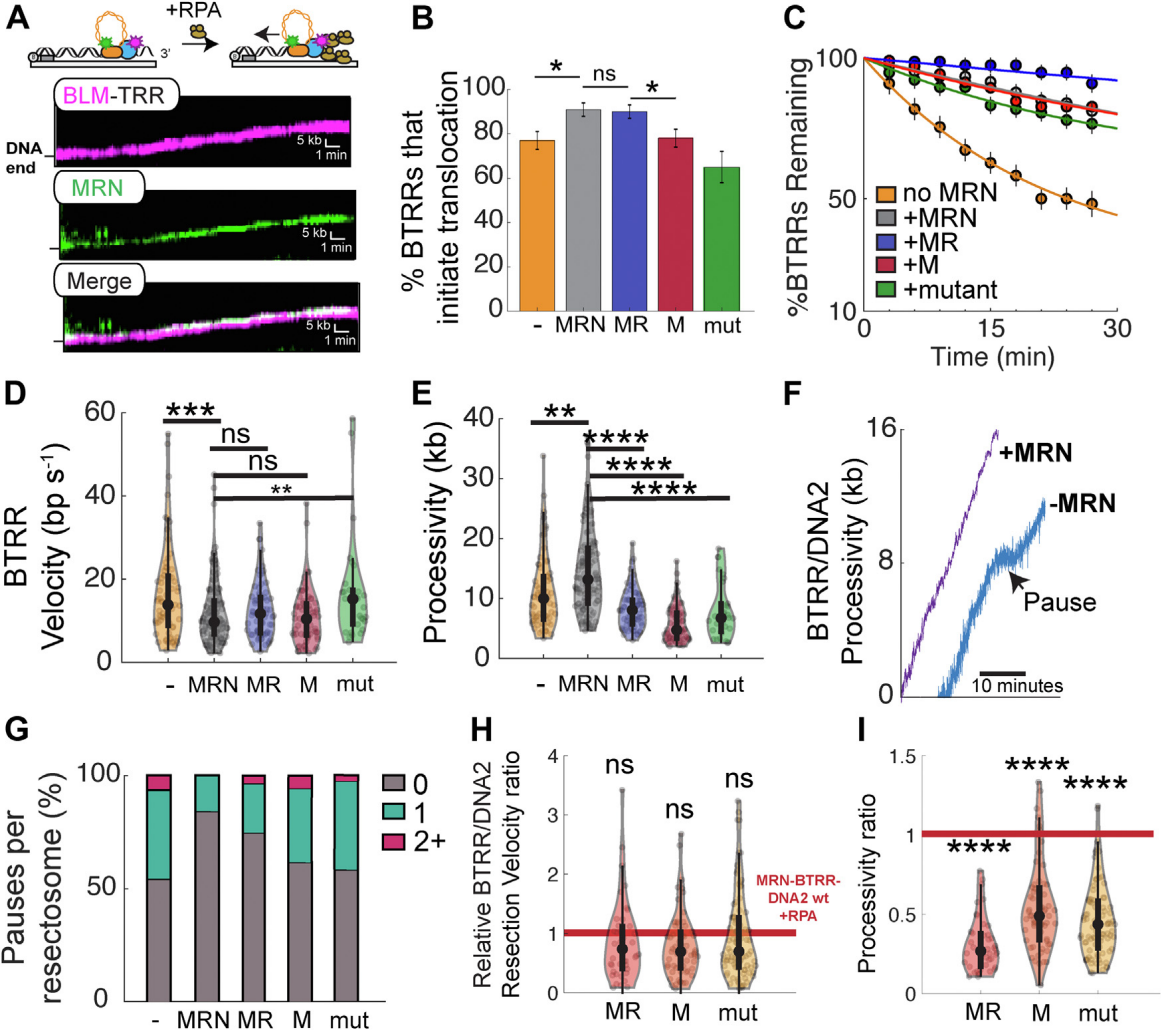
**MRN prevents BTRR DNA dissociation.***A*, representative kymographs showing the helicase activity of BLM (*magenta*) with MRN (*green*) and the TOP3–RMI1/2 complex (*dark*) along DNA. *B*, helicase initiation by the BTRR complex with the indicated MRN subcomplexes in the presence of RPA. The MRN mutant (mut) encodes RAD50(S1202R). n > 40 molecules for all conditions measured across at least two flow cells. Error bars represent SD as determined by bootstrap analysis. *C*, MR is sufficient to retain the BTRR complex on DNA. In the presence of MR(N), BTRR rarely dissociates from the DNA during the 30 min experiment. *D*, velocities and (*E*) processivities of the BTRR complex with the indicated MRN variants. *F*, Representative particle tracking of BTRR/DNA2 resection with (*purple*) and without MRN (*blue*). *G*, MRN decreases pausing events during BTRR–DNA2 resection. *H* and *I*, relative resectosome velocities and processivities with the indicated MRN mutants. The velocity and processivity are normalized to wildtype resectosome complexes. n > 40 molecules for all conditions measured across at least two flow cells (ns, *p* > 0.05; ∗*p* < 0.05; ∗∗*p* < 0.01; ∗∗∗*p* < 0.001; ∗∗∗∗*p* < 0.0001). BLM, bloom syndrome helicase; BTRR, BLM–TOP3A–RMI1/2; MR, MRE11–RAD50; MRN, MRE11–RAD50–NBS1; ns, not significant; RPA, replication protein A; TOP3, topoisomerase IIIa.

Next, we investigated how TOP3A–RMI1/2 regulates BLM helicase. BLM was fluorescently labeled *via* a fluorescent anti-hemagglutinin (HA) antibody directed to an N-terminal HA epitope, as we described previously (50). TOP3A–RMI1/2 colocalized with BLM at the free DNA ends (Fig. 3*A*). We recently showed that RPA aids BLM in initiating helicase activity from 3′-ssDNA overhangs (50). When RPA is omitted, only ∼30% of BLM molecules initiate translocation. However, adding TOP3A–RMI1/2 increases the number of translocating BLM molecules ∼2.3-fold (77%; n = 86/111) (Fig. 3*B*). TOP3A was sufficient to recapitulate most of this stimulation (62%; n = 83/133), whereas adding RMI1/2 alone slightly decreased the number of translocating BLM molecules (19%; n = 34/182). Finally, adding RPA did not further stimulate the number of translocating BTRR complexes (83%; n = 67/81). Surprisingly, although TOP3A–RMI1/2 initiated more BLM helicases, it also decreased the velocity of BLM approximately two-fold (14 ± 11 bp s^−1^; n = 86) and slightly reduced processivity ∼1.2-fold (14 ± 8 kb) (Fig. 3, *C* and *D* and Table S3). Furthermore, >50% of BTRR molecules dissociate from DNA during the 30 min experiment (Fig. 4*C*). BLM–TOP3A had a similar velocity and processivity as the BTRR complex (n = 83). However, the processivity of BLM–RMI1/2 was further reduced approximately two-fold compared with the BTRR complex (8 ± 3 kb; n = 34). Adding RPA did not change BTRR velocity but slightly decreased the average processivity (11 ± 6 kb; n = 67). We conclude that TOP3A–RMI1/2 helps to initiate DNA unwinding but reduces the velocity and processivity of BLM.

### MRN prevents BTRR dissociation from DNA

Having established that TOP3A–RMI1/2 helps initiate DNA unwinding, we tested whether MRN further stimulates the BTRR complex. MRN is important for BLM recruitment to DSB and has been shown to stimulate unwinding activity (30, 60, 61). As expected, MRN colocalizes with BTRR and moves together with BTRR during DNA unwinding (Fig. 4*A*). In the presence of 1 nM RPA, ∼90% (n = 131/145) of MRN–BTRR resectosomes initiated DNA unwinding (Fig. 4*B*). MRN also retained BLM molecules on the DNA (Fig. 4*C*). In the presence of MRN, fewer than 30% of the BLM molecules dissociated from the DNA (n = 131). This resulted in a 1.3-fold increase in processivity (14 ± 7 kb; n = 131) but a ∼1.4-fold decrease in MRN–BTRR helicase velocity (12 ± 8 bp s^−1^; n = 131) (Fig. 4, *D* and *E*).

To better understand the effect of MRN on BLM activity, we repeated the helicase assays with MR and MRE11 subunits (Fig. 4, *B*–*E*). MR was sufficient to initiate the helicase activity of BLM but decreased processivity. MRE11 alone did not stimulate BLM initiation, suggesting the RAD50 subunit is critical for regulating the activity of BLM. To test whether the ATPase of MRN is also required to stimulate BLM, we repeated helicase experiments with the ATPase-deficient MR(S1202R)N (62, 63). Interestingly, MR(S1202R)N decreased both BLM helicase initiation and processivity. *In vitro* pulldown experiments showed that BLM interacts with both NBS1 and RAD50; MRE11 did not interact with BLM (Fig. S3*A*). MRN and BLM may also interact indirectly *via* RPA and/or TRR (64). We conclude that the ATPase of MRN activity is important for stimulating BLM, possibly by initial DNA unwinding and/or promoting DNA tethering at the ssDNA/dsDNA junction.

### MRN synchronizes the BLM and DNA2 motors

The unwinding rate of DNA2 is approximately three-fold slower than that of BLM in the presence of RPA (Fig. 2*F*). The yeast homolog of BLM, Sgs1, and Dna2 also show approximately two-fold difference in unwinding rates (65). This difference in unwinding rates of BLM and DNA2 can lead to discoordination between the two motors. Consistent with this notion, we observed that ∼45% of the BTRR–DNA2 resectosomes paused for >30 s during DNA resection (n = 42/94) with 12% (n = 5/42) of these complexes pausing two or more times during their resection trajectories (Fig. 4, *F* and *G*). The change in resection velocity after the pause was heterogeneous and did not correlate with the prepause velocity (Fig. S3*E*). Adding MRN suppressed these pauses (83% of resectosomes did not pause; n = 68/82). MRN also suppresses pauses with the minimal MRN–BLM–DNA2 assembly (83% did not pause; n = 25/30). MR and MRE11 did not stimulate resection and suppress pausing (Fig. 4, *G*–*I*). Interestingly, MR(S1202R)N decreased processivity, velocity, and could not suppress pausing (Fig. 4, *G*–*I*). Thus, the ATP-dependent activities of MRN, along with BLM and DNA2, are also required to promote efficient DNA resection, possibly by stimulating the engagement of BLM with the ss/ds junction. We also tested whether pausing was sequence or GC content specific. The GC content of λ-DNA is greater on the *cosL* side than on the *cosR* side of our DNA substrate (Fig. S3*B*). Therefore, we assayed resection from the GC-rich (*cosL*) end or GC-poor (*cosR*) ends. The pausing frequency was similar on both substrates, suggesting that pausing is not strongly sequence or GC content dependent but instead may be caused by the accumulation of ssDNA (Fig. S3, *C* and *D*). We conclude that MRN coordinates BLM and DNA2 to stimulate efficient DNA resection.

## Discussion

**Figure 5:**
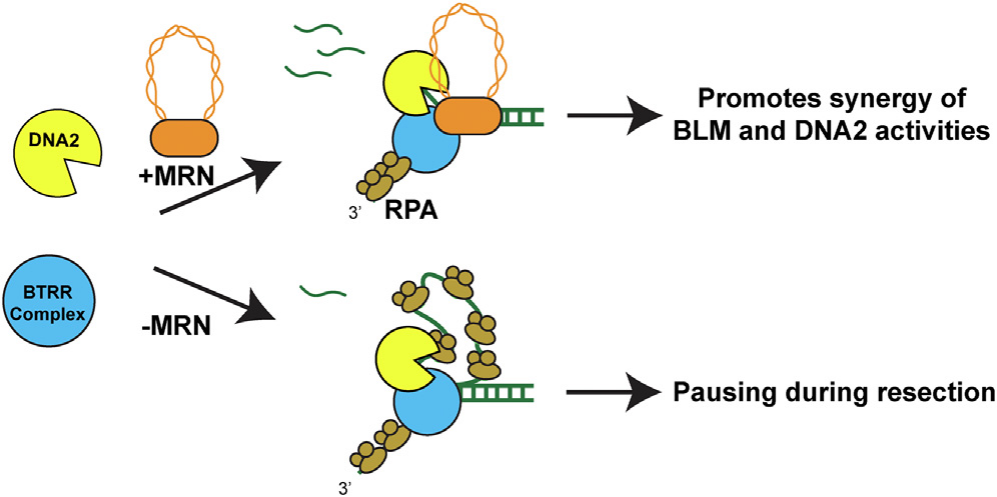
**Model of how MRN regulates DNA resections**.

DNA resection is catalyzed by either the EXO1 or DNA2 nucleases. Although EXO1 may be the predominant resection nuclease in human cells (48, 66, 67, 68), DNA2 is better at processing apurinic/apyrimidinic sites and 8-oxoguanines (69). Furthermore, a super-resolution imaging study found comparable recruitment of both DNA2 and EXO1 at induced DSBs, suggesting that both nucleases are required during DNA resection (61). We had previously shown that MRN and BLM act as processivity factors for EXO1 in the presence of RPA (28, 50). We expand on the earlier study to show that TOP3A–RMI1/2 does not stimulate BLM–EXO1 resection. Instead, TOP3A–RMI1/2, in combination with MRN, stimulate DNA2-mediated resection. In addition, MRN plays a scaffolding role by assembling the resectosome at a DSB and suppressing the dissociation of BTRR from DNA. MRN also prevents pausing by coordinating BLM and DNA2 during DNA resection (Fig. 5).

BLM can either processively unwind DNA or strand switch between the Watson or Crick ssDNA strands (50, 70, 71, 72). To promote processive movement and suppress strand-switching, BLM must engage the ss/dsDNA junction rather than the ssDNA formed during unwinding (73, 74). MRN recognizes both dsDNA and ssDNA/dsDNA junctions and makes protein–protein interactions with BLM through both the RAD50 and NBS1 subunits (Fig. S3*A*). We propose that these physical interactions with BLM increase the processivity of BLM and suppress pausing by anchoring BLM to the ss/ds DNA junction. Similarly, the TRR complex also binds free DNA ends and interacts with BLM (34). Thus, TRR may also prevent BLM from engaging partially unwound DNA to promote processive helicase activity.

Here, we show that DNA resection requires the concerted activity of motors of both BLM and DNA2. However, the helicase activity of DNA2 is only detectable when its nuclease is disabled *in vitro* (54, 56). How is DNA2 then able to use its helicase activity within the resectosome? One possibility is that a physical interaction between BLM and DNA2 stimulates the helicase of DNA2 and/or suppresses its nuclease activity. BLM and DNA2 physically interact and colocalize at DNA ends *in vitro* and in cells (30, 50, 61, 75). WRN also interacts with DNA2 *via* its helicase domain, which is conserved in BLM (45). Future studies will be required to further map this interaction and its significance for regulating both motors. BLM may also promote the helicase of DNA2 by providing a long 5′-ssDNA overhang that engages that domain. The structure of mouse DNA2 reveals that ssDNA threads through a narrow protein channel that requires >10 nt to interact with both the nuclease and helicase domains. In the absence of such ssDNA, the nuclease activity may degrade ssDNA that cannot thread into the helicase domain (55). However, the unwinding of the long ssDNA flap by BLM may overcome the inhibition by the nuclease domain to engage the helicase and provide stimulation of both motors. This is consistent with our results and previous *in vitro* and *in vivo* data that showed that hd BLM and its yeast homolog, Sgs1, downregulate DNA resection (19, 20, 30, 32, 50).

Pausing during DNA resection has been observed with the bacterial RecBCD nuclease–helicase complex following recognition of the recombination hot spot sequence χ (crossover hot spot instigator-Chi) (76, 77, 78). RecBCD is functionally reminiscent of BTRR–DNA2 because it also encodes a fast (RecD) and slow (RecB) motor of opposite polarity. Two motors that move along opposite DNA strands with different speeds will generate a long ssDNA loop between them. Such loops are generated by the *Escherichia coli* RecBCD helicase–nuclease because of differences in the translocation rates between the RecD and RecB motors (79). The molecular origin of RecBCD pausing stems from the slower motor “catching up” with the faster motor because of a conformational switch after χ recognition.

The underlying reason for pausing by BTRR–DNA2 is unknown, but it is unlikely to depend on a χ-like DNA sequence as sequence-dependent resection regulation has not been observed in yeast and human resectosomes. We conjecture that BTRR may unwind DNA in front of DNA2, leading to a growing ssDNA loop that ultimately pauses the entire complex. In this model, MRN prevents the accumulation of such ssDNA loops by synchronizing BLM and DNA2 helicase velocities. In support of this hypothesis, a recent single-molecule study showed that BLM retains contact with ssDNA as it unwinds dsDNA (75). Additional high-resolution electron microscopy and other biochemical studies will be required to directly image an ssDNA loop between BLM and DNA2.

MRE11 and BLM colocalize early at DNA breaks immediately following damage in cells (61). This is consistent with our results showing that MRN assembles the resectosome at a DNA break. However, MRE11 and BLM do not associate as closely in the later stages of DNA resection (61). The role of MRN may thus be critical in the initiation and early coordination of BLM and DNA2. In the later stages of DNA resection, pausing may act as a negative DNA resection signal. Such pauses slow resection, possibly limiting over-resection and giving RAD51 sufficient time to complete the homology search. Together, this work shows that its conserved accessory factors regulate the helicase activity of BLM and that coordination with MRN and DNA2 stimulates DNA resection and, ultimately, efficient HR.

## Experimental procedures

### Protein cloning and purification

Oligonucleotides were purchased from IDT. Human RPA (pIF47) was purified from *E. coli* using a pET expression vector (48, 80, 81). Epitope-tagged human EXO1 (pIF7) and MRN (pIF926) were purified from insect cells as previously described (28, 48, 50, 52, 82). FLAG-tagged human DNA2 nuclease–deficient (D277A) and hd (K654R) mutants were generated by Phusion site-directed mutagenesis (Thermo Fisher) of wildtype DNA2-FLAG (pIF494) using oligos MS0015 and MS0016 for D277A (pIF495) and MS0017 and MS0018 for K564R (pIF496) (Table S4). Wildtype DNA2, DNA2 (D277A), and DNA2 (K654R) were purified from insect cells as previously described (50, 82).

For single-molecule fluorescent imaging, 3xHA-BLM-His_6_ (pIF527) was expressed in Sf21 insect cells infected using the Bac-to-Bac expression system (Life Tech) (83). Cells were harvested 72 h after infection, pelleted, frozen, and stored at −80 °C. Cells were homogenized in buffer A containing 50 mM Tris–HCl (pH 7.5), 500 mM NaCl, 10% glycerol, 2 mM β-mercaptoethanol, 10 mM imidazole, and 250 mM PMSF in a Dounce homogenizer (Kimble Chase; Kontes) followed by sonication on ice. Insoluble material was pelleted for 1 h at 100,000*g*, and the supernatant was added to nickel–nitrilotriacetic acid (Ni–NTA) resin (QIAGEN, catalog no.: 30410) in batch and eluted with an imidazole gradient containing 50 mM Tris–HCl (pH 7.5), 500 mM NaCl, 10% glycerol, 2 mM β-mercaptoethanol, and 10 to 250 mM imidazole. BLM fractions were then loaded on a 1 ml HiTrap Heparin (GE Healthcare) and eluted with a gradient from buffer B (50 mM Tris–HCl [pH 7.5], 100 mM NaCl, 10% glycerol, and 1 mM DTT) to buffer C (50 mM Tris–HCl [pH 7.5], 1 M NaCl, 10% glycerol, and 1 mM DTT). BLM was further purified using a Superose 6 in buffer D (50 mM Tris–HCl [pH 7.5], 200 mM NaCl, 10% glycerol, and 1 mM DTT).

pRSF-Duet plasmids containing His_6_-TOP3A and RMI1-His_6_/FLAG-RMI2 were kindly provided by Patrick Sung and Jim Daley. The TOP3A plasmid was transformed into Rosetta (DE3) pLysS cells and grown in LB media and induced with 0.2 mM IPTG for 18 h at 16 °C. Cells were pelleted and resuspended in buffer E (50 mM Tris–HCl [pH 7.5], 1 M KCl, 10% glycerol, and 1 mM DTT) supplemented with 0.01% Igepal and 1 mM PMSF. The cells were sonicated, and the soluble material was clarified by centrifugation at 35,000 RCF for 45 min. The supernatant was added to Ni–NTA in batch, washed with buffer A supplemented with 20 mM imidazole, and eluted with buffer F (50 mM Tris–HCl [pH 7.5], 200 mM KCl, 10% glycerol, and 1 mM DTT) supplemented with 250 mM imidazole. TOP3A was further purified using a 1 ml HiTrap SP (GE Healthcare) with a gradient from buffer G (50 mM Tris–HCl [pH 7.5], 50 mM KCl, 10% glycerol, and 1 mM DTT) to buffer H (50 mM Tris–HCl [pH 7.5], 1 M KCl, 10% glycerol, and 1 mM DTT) and dialyzed overnight at 4 °C in buffer I (50 mM Tris–HCl [pH 7.5], 200 mM KCl, 10% glycerol, and 1 mM DTT).

The RMI1-His_6_/FLAG-RMI2 plasmid was transformed into Rosetta (DE3) pLysS cells and grown in LB media and induced with 0.2 mM IPTG for 18 h at 16 °C. The cells were sonicated, centrifuged, and purified by Ni–NTA resin similar to TOP3A. RMI1/2 elution from Ni–NTA purification was diluted to 50 mM KCl with buffer J (50 mM Tris–HCl [pH 7.5], 10% glycerol, and 1 mM DTT), loaded on a 5 ml HiTrap Q XL (GE Healthcare), and eluted with a gradient with buffers G and H. RMI1/2 was further purified using a Superdex 200 Increase (GE Healthcare) in buffer I. The TOP3A–RMI1/2 complex was assembled by incubating purified TOP3A and RMI1/2 (1:3 ratio) followed by purification using a Superdex 200 Increase in buffer I.

### Single-molecule fluorescence microscopy

All single-molecule data were collected on a Nikon Ti-E microscope in a prism-total internal reflection fluorescence configuration equipped with a prior H117 motorized stage. Flow cells were loaded into a custom-designed stage insert incorporating a chip mount, fluidic interface, and heating element (49). All experiments were maintained at 37 ˚C by a combination of an objective heater (Bioptechs) and a custom-built stage-mounted heating block. The flow cell was illuminated with a 488 nm laser (Coherent) through a quartz prism (Tower Optical Co). Data were collected with a 200 ms exposure, 2 s shutter (Vincent Associates) resulting in 1800 frames in 1 h, through a 60× water-immersion objective (1.2 numerical aperture; Nikon), a 500 nm long pass (Chroma), and a 638 nm dichroic beam splitter (Chroma), which allowed two-channel detection through two EMCCD cameras (Andor iXon DU897; cooled to −80 °C). Images were collected using Nikon NIS-Elements software and saved in an uncompressed TIFF file format for later analysis (see later).

DNA substrates for single-molecule studies contained a 78 nt 3′-overhang or 12 nt 5′-overhang. These were prepared by annealing oligonucleotides IF007 and LM003 (3′-overhang ligated to cosR site), IF006 and LM024 (3' overhang ligated to cosL site), or IF007 only (5′-overhang) (Table S4) (28).

In our imaging buffer (40 mM Tris [pH 8.0], 60 mM NaCl, 200 μg/ml bovine serum albumin [BSA], 2 mM DTT, 2 mM MgCl_2_, and 1 mM ATP), we typically observe intermittent fluorescent emission (blinking), which is an intrinsic property of single QDs (84). These blinking events indicate that our fluorescent trajectories are from an individual QD because of the unlikely situations of two QDs blinking simultaneously.

### Particle tracking analysis

The image stacks collected from the EMCCD cameras were exported as full-resolution TIFF stacks. To correct for XY-stage sample drift, a stationary particle on the flow cell surface was picked, and its position was tracked by fitting the point-spread function to a 2D-Gaussian using a custom-written ImageJ script (available at: https://github.com/finkelsteinlab/single-particle-tracking-scripts). XY drift was then subtracted from all resectosome complexes during postprocessing. For each frame, the point spread function of DNA-bound proteins was fit to a 2D Gaussian to obtain (*x*, *y*) coordinates with subpixel resolution. We ensured that resectosome components were DNA bound by briefly stopping buffer flow at the beginning of each experiment. Stopping buffer flow recoils the DNA and all associated proteins to the diffusion barrier, providing a useful control that these are not surface-tethered particles. Only DNA-bound particles were included in all subsequent analyses. We did not attempt to analyze the trajectories of particles that moved less than 1 kb, which is approaching the resolution of the DNA curtain assay under the buffer flows used here (∼500 bp).

Particle trajectories were analyzed in MATLAB R2018a-version (MathWorks). For individual moving particles, the processivity was determined by measuring the distance traveled along DNA, and velocity was determined by fitting the time-dependent position along DNA to a line. To determine DNA binding lifetimes, we measured the time each molecule was bound to DNA. The survival probabilities were fit to a single exponential decay in MATLAB. Particles from at least two flow cells were pooled for the final analysis. Statistical significance was determined *via* the Student’s *t* test.

### Fluorescent protein labeling

3xHA-BLM (40 nM) were conjugated to QDs preincubated with a rabbit anti-HA antibody (ICL Lab) on ice for 10 min in 20 μl. Next, BLM was incubated with the anti-HA QDs at a ratio of 1:2 for an additional 10 min on ice, diluted with imaging buffer (40 mM Tris [pH 8.0], 60 mM NaCl, 200 μg/ml BSA, 2 mM DTT, 2 mM MgCl_2_, and 1 mM ATP) to 200 μl and injected into the flow cell. FLAG-TOP3A–RMI1/2 (80 nM) or FLAG-DNA2 (40 nM) were labeled with QDs preincubated with a mouse anti-FLAG antibody (Sigma–Aldrich) on ice for 10 min prior to injection. In addition, biotin–MRN (2 nM) was labeled *via* streptavidin QDs. Saturating biotin was added to the protein–QD conjugates to bind free streptavidin sites prior to injection.

### Quantification and statistical analysis

For Figure 1, Figure 2, Figure 3, Figure 4, n represents the number of molecules. Quantification and statistical analyses were done using MATLAB (version: R2018a). Fluorescent particles were tracked using an in-house ImageJ script (available at https://github.com/finkelsteinlab/single-particle-tracking-scripts) where the positions of individual molecules on DNA were determined by fitting the point spread function to a 2D Gaussian. Trajectories were used to calculate the velocity and processivity for BLM and DNA resection complexes. Statistical details of experiments can be found in the Results section and figure legends where indicated.

## Data availability

All custom MATLAB and FIJI scripts are available at https://github.com/finkelsteinlab/single-particle-tracking-scripts.

## Contact for reagent and resource sharing

Further information and requests for resources and reagents should be directed to and will be fulfilled by the Lead Contact, Ilya Finkelstein (ilya@finkelsteinlab.org).

## Supporting information

This article contains supporting information.

## Conflict of interest

The authors declare that they have no conflicts of interest with the contents of this article.
