# Supplementary material for: The MRN complex and topoisomerase IIIa–RMI1/2 synchronize DNA resection motor proteins
Source: J Biol Chem. 2022 Dec 16;299(2):102802. doi: 10.1016/j.jbc.2022.102802 (PMC9971906; doi:10.1016/j.jbc.2022.102802)
Supplement: Supporting information [file mmc1.pdf]

SUPPLEMENTAL MATERIAL

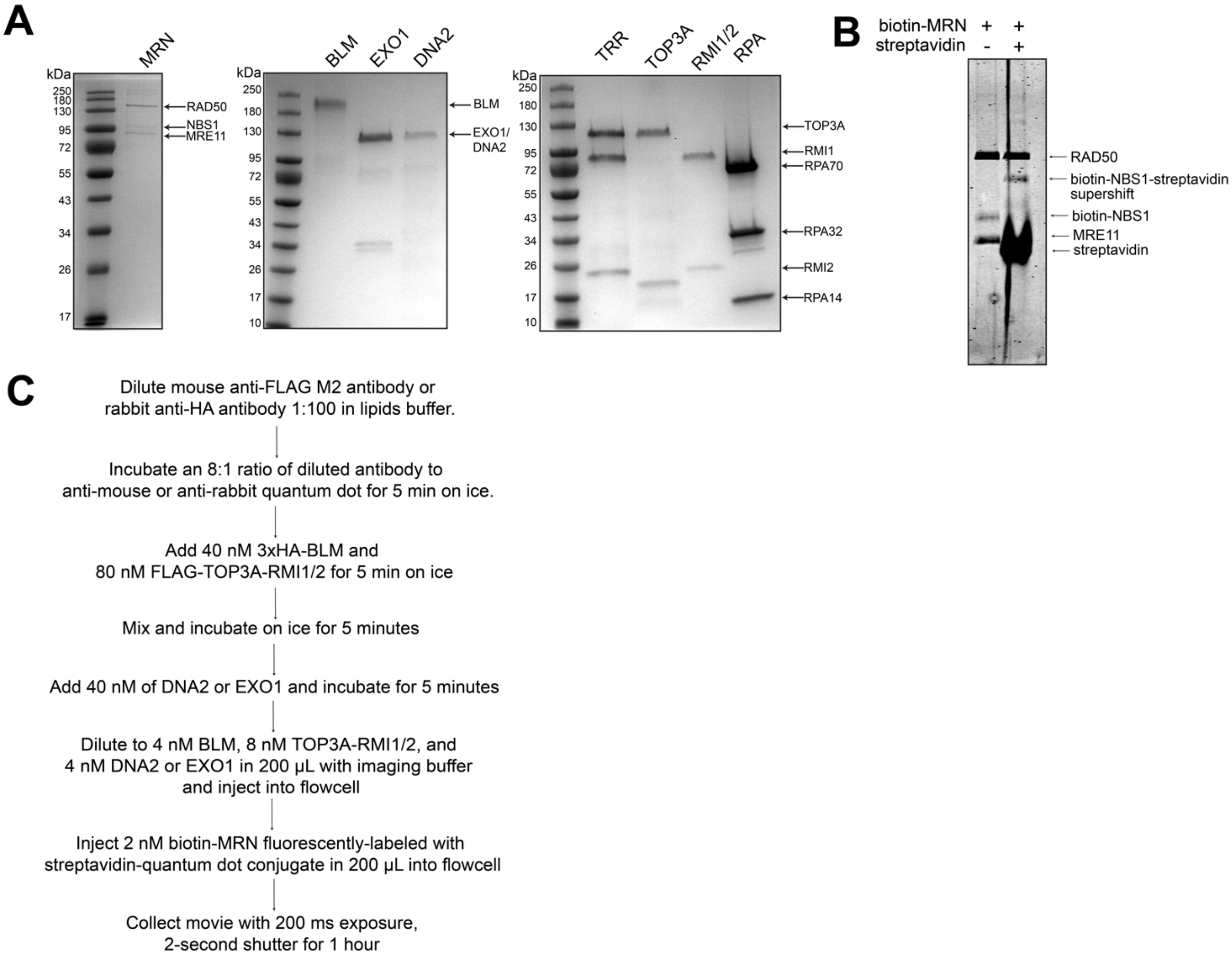

Supplemental Figure 1. (A) SDS-PAGE gels of the recombinant proteins used in this study. **(B)** SDS-PAGE gel showing biotin-MRN and biotin-MRN + streptavidin. The samples were not boiled to preserve the biotin-streptavidin interactions. The upshifted biotin-NBS1-streptavidin conjugates are indicated. The complete disappearance of the biotin-NBS1 band indicates that nearly 100% of the purified MRN is biotinylated. **(C)** Flowchart describing the single-molecule resection experiments.

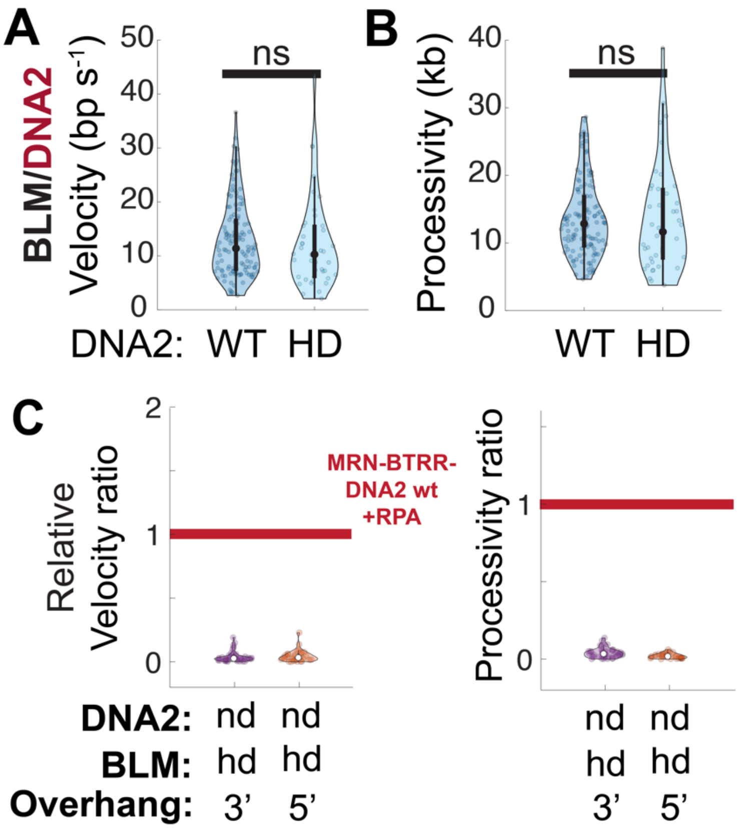

Supplemental Figure 2. (A) Velocities and **(B)** processivities of the wild type (WT) BLM/DNA2 (n=126), and the helicase-deficient (HD) DNA2(K654R) mutant (n=42). Black bars show the interquartile range (thick bars) and 1.5x interquartile range (thin bars). The black dot in the middle is the median **(C)** Ratio of MRN/BTRR/DNA2 velocities (left) and processivities (right) with nuclease-deficient (nd) DNA2 and helicase-deficient (hd) BLM mutants (n>30 for both conditions from two flowcells). Both velocity and processivity are normalized to the corresponding values for the WT MRN/BTRR/DNA2 complex (red line). (not significant; ns, p>0.05).

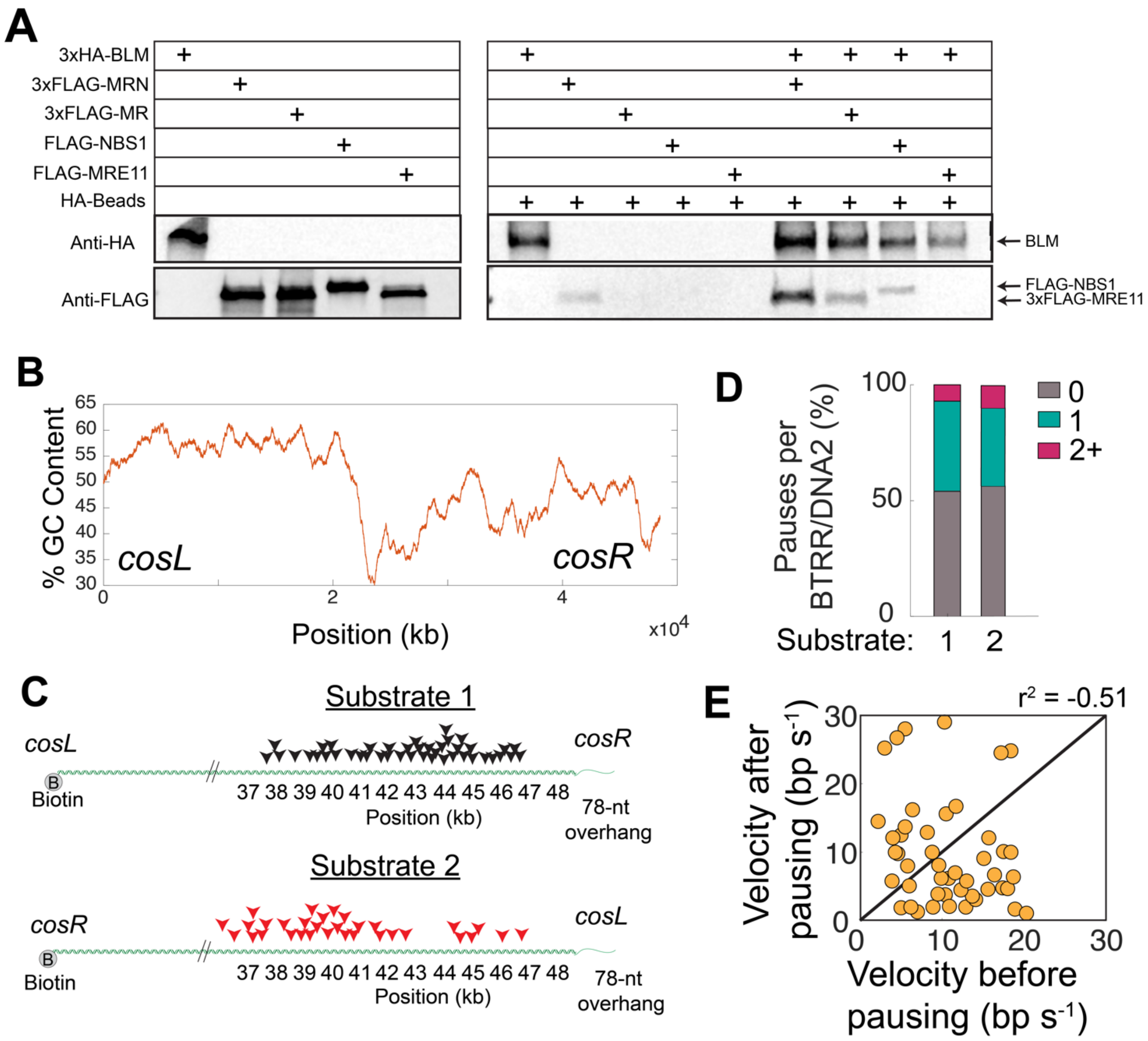

Supplemental Figure 3. (A) *In vitro* pulldown assays show that BLM interacts with NBS1 and RAD50. **(B)** The DNA substrate GC content is higher near *cosL* relative to *cosR*. The substrate is derived from λ-phage DNA. The **(C)** Pausing positions for both DNA substrates (n=42 and 35 pauses for substrates 1 and 2, respectively). **(D)** Pausing frequency per resectosome for the indicated substrates. **(E)** Velocities of individual BTRR/DNA2 complexes before and after pausing. Dashed line is shown as a reference with a slope of m = 1 (n=42 resectosomes).

Table S1: **Velocity and processivity for DNA2 and EXO1-mediated DNA resection**

Sample	Processivity (kb) mean ± st. dev.	Velocity (bp s^{-1}) mean ± st. dev.	Number of molecules (n)
BLM/DNA2	13 ± 6	13 ± 7	126
MRN/BLM/DNA2	12 ± 5	9 ± 6	30
BTRR/DNA2	13 ± 5	9 ± 6	94
MRN/BTRR/DNA2	18 ± 6	18 ± 11	82
BLM/EXO1	15 ± 7	13 ± 9	124
MRN/BLM/EXO1	14 ± 6	12 ± 7	82
BTRR/EXO1	12 ± 8	14 ± 11	79
MRN/BTRR/EXO1	12 ± 7	13 ± 10	57
MRN/BTRR/DNA2 (D277A)	2 ± 2	2 ± 2	89
MRN/BTRR/DNA2 (K654R)	13 ± 6	11 ± 7	76
MRN/BLM (K695A)/TRR/DNA2 (D277A) + 3'-overhang	0.7 ± 0.6	0.7 ± 0.7	44
MRN/BLM (K695A)/TRR/DNA2 (D277A) + 5'-overhang	0.4 ± 0.3	0.8 ± 0.8	32
MR/BTRR/DNA2	6 ± 3	15 ± 12	42
Mre11/BTRR/ DNA2	10 ± 5	14 ± 10	63
MRN(S1202R)/BTRR/ DNA2	8 ± 4	17 ± 13	87
BLM/DNA2 HD	14 ± 8	12 ± 8	42

Table S2: **Velocity and processivity for DNA2 helicase activity**

**Sample**	**Processivity (kb)** mean ± st. dev[	**Velocity (bp s^{-1})** mean ± st. dev[	**Number of molecules (n)**
DNA2 (D277A)	4 ± 2	5 ± 3	23
MRN/DNA2 (D277A)	3 ± 1	3 ± 1	19
TRR/DNA2 (D277A)	4 ± 2	3 ± 2	27
MRN/TRR/ DNA2 (D277A)	4 ± 1	4 ± 2	15

Table S3: **Velocity and processivity for BLM helicase activity**

Sample	Processivity (kb) mean ± st. dev.	Velocity (bp s^{-1}) mean ± st. dev.	Number of molecules (n)
BLM	17 ± 7	25 ± 18	90
BTRR	14 ± 8	14 ± 11	86
BLM/TOP3A	13 ± 8	13 ± 9	83
BLM/RMI1/2	8 ± 3	10 ± 6	34
BTRR/RPA	11 ± 6	17 ± 11	67
MRN/BTRR/RPA	14 ± 7	12 ± 8	131
MR/BTRR/RPA	8 ± 3	13 ± 7	68
Mre11/BTRR/RPA	6 ± 3	11 ± 7	66
MRN(S1202R)/BTRR/RPA	8 ± 5	17 ± 13	30

Table S4: **Oligonucleotides used in this study.**

Name	Sequence
IF006	[p]GGGCGGCGACCT[Bio]
IF007	[p]AGGTCGCCGCCC[Bio]
LM003	[p]GGGCGGCGACCT<br>TTTTTTTTTTTTTTTTTTTTTTTTTTTTTTTTTTTTTTTTTTTTTTTTT<br>TTTTTTTTTTTTTTTTTTTTTTTTTTTTT
LM024	[p]AGGTCGCCGCCC<br>TTTTTTTTTTTTTTTTTTTTTTTTTTTTTTTTTTTTTTTTTTTTTTTTT<br>TTTTTTTTTTTTTTTTTTTTTTTTTTTTT
MS0015	TATTTTGCCTTTCAATCCAAACCTAGG
MS0016	GCCGTTACAGTTGGTGTGAAAATACATCGAG
MS0017	TCCTGTCCCAGGCATACCC
MS0018	CGTACAACTACGATATGTACTCTCGTAAGAATTC
